# Manufacturing and Characterization of Environmentally Friendly Wood Plastic Composites Using Pinecone as a Filler into a Bio-Based High-Density Polyethylene Matrix

**DOI:** 10.3390/polym13244462

**Published:** 2021-12-20

**Authors:** Maria del Carmen Morcillo, Ramón Tejada, Diego Lascano, Daniel Garcia-Garcia, David Garcia-Sanoguera

**Affiliations:** Technological Institute of Materials (ITM), Universitat Politècnica de València (UPV), Plaza Ferrándiz y Carbonell 1, 03801 Alcoy, Spain; mamores@epsa.upv.es (M.d.C.M.); rateol@epsa.upv.es (R.T.); dielas@epsa.upv.es (D.L.); dagarsa4@epsa.upv.es (D.G.-G.)

**Keywords:** natural fillers, mechanical properties, wood plastic composite, pinecone, polyethylene

## Abstract

The use of wood plastic composites (WPC) is growing very rapidly in recent years, in addition, the use of plastics of renewable origin is increasingly implemented because it allows to reduce the carbon footprint. In this context, this work reports on the development of composites of bio-based high density polyethylene (BioHDPE) with different contents of pinecone (5, 10, and 30 wt.%). The blends were produced by extrusion and injection-molded processes. With the objective of improving the properties of the materials, a compatibilizer has been used, namely polyethylene grafted with maleic anhydride (PE-g-MA 2 phr). The effect of the compatibilizer in the blend with 5 wt.% has been compared with the same blend without compatibilization. Mechanical, thermal, morphological, colorimetric, and wettability properties have been analyzed for each blend. The results showed that the compatibilizer improved the filler–matrix interaction, increasing the ductile mechanical properties in terms of elongation and tensile strength. Regarding thermal properties, the compatibilizer increased thermal stability and improved the behavior of the materials against moisture. In general, the pinecone materials obtained exhibited reddish-brown colors, allowing their use as wood plastic composites with a wide range of properties depending on the filler content in the blend.

## 1. Introduction

In the past years, social awareness has significantly increased derived from environmental issues related to the generation of wastes, petroleum shortage, and the increasing need to reduce the carbon footprint [1]. The worldwide plastic production is nowadays about 300 Mt/year, verifying the great amount of wastes that plastic industry produces [2].

In this context, bio-based polymers can successfully reduce the use of fossil resources through the use of biomass or renewable resources, at the same time that they reduce the carbon footprint [3]. Within plastic industry, high density polyethylene (HDPE) is one of the most utilized commercial plastics, right after polyvinyl chloride (PVC) and polypropylene (PP) in terms of production volume [4]. This is the reason for which BioHDPE or “green-polyethylene” is a good solution for reducing the problems related to fossil resources. This BioHDPE is produced by polymerization of ethylene obtained from catalytic dehydration of bioethanol. BioHDPE has the same physical properties as its petrochemical counterpart (HDPE), which possesses good mechanical resistance, high ductility, and improved waterproof capabilities [5,6]. In 2018, bio-based polyethylenes represented approximately 9.5% of the global bioplastic production, reaching almost 200,000 tons/year [7]. Normally, BioHDPE injected pieces can be used to produce either rigid parts or flexible films and packages [6].

Natural fillers have been used for a long time with the objective of reducing the cost of material, in most of the cases the introduction of these fillers in a limited amount does not affect significantly the properties of the composite. So, it is common to introduce the maximum possible amount of filler in order to reduce the price of the material as much as possible [8].

In addition to the use of polymers from renewable sources, the interest for natural filler reinforced plastics (NFRP) [9,10,11] and wood plastic composites (WPC) [12,13,14], has risen. In this context, this kind of cellulosic reinforcements positively contribute to the obtention of environmentally friendly materials, reduce the cost of biopolymers, and upgrade industrial and agroforestry wastes [15]. In this sense, these materials can also directly focus on reducing environmental limitations, which are product of a linear economy, thus, allowing to reuse different wastes in order to enhance the concept of circular economy [16]. This transition from “waste elimination” to “waste reutilization as added-value materials” is the key to reach a circular economy [17]. In the last decades, fillers and natural residues have been widely used in polymeric compounds [18,19]. These fillers can be obtained from minerals, animals, or plants. However, fillers obtained from plants, either particles or fibers, are the most common to be used in green composites [20,21]. This is due to them being obtainable from agricultural and industrial wastes or by-products from food processing. This allows to improve the value of discarded materials and encourage circular economy [22]. Following this concept, in recent years, new materials have been developed, based on vegetal wastes such as orange peel flour [23], babassu shell flour [24], almond shell flour [25], or fibers like pinecone leaf fiber [26], jute fiber [27], banana stem fiber [28], coconut fiber [29], flax fiber [30], etc.

In this investigation work, environmentally friendly composites based on a polymeric matrix of BioHDPE with pinecone particles have been obtained and analyzed. The compatibilizer PE-g-MA was introduced with the objective of improving the interaction between the fibers and the matrix and upgrading the final properties of the green composites. Mechanical, morphological, thermal, thermomechanical, and wettability properties of the materials were characterized. The main objective of this work is to validate the development of a bio-based composite obtained with a natural filler of great abundance such as pinecone and the implication of the use of commercial compatibilizers on the performance of the resulting composite material. In addition, the aim is to maximize the load content of pinecone in order to minimize the cost of the resulting composite material without reducing its main performances. It is also intended to meet the requirements for use as a wood plastic composite in the home furnishing sector with a wider range of properties, compared to those currently used in the market.

## 2. Materials and Methods

### 2.1. Materials

Bio-HDPE used was SHA7260 grade, supplied in pellets formed by FKuR Kunststoff GmbH (Willich, Germany) and manufactured by Braskem (São Paulo, Brazil), with a density of 0.955 g·cm^−3^ and a melt flow index (MFI) of 20 g/10 min (190 °C/21.2 N). Polyethylene-grafted maleic anhydride (PE-g-MA) with CAS Number 9006-26-2 and MFI values of 5 g/10 min (190 °C/21.2 N), were supplied by Sigma-Aldrich S.A. (Madrid, Spain) and it was selected due to their great functionality.

**Figure 1:**
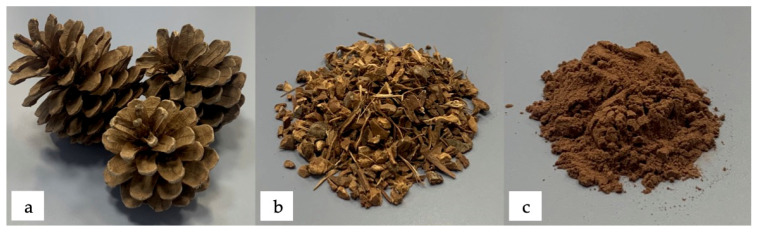
Visual aspect of pinecone: (**a**) unprocessed; (**b**) crushed; (**c**) ground and sieved.

Pinecone used was obtained from Pinus Halepensis, which is a pine native from Mediterranean region (Figure 1a). Pinecone filler was prepared in two stages, first was crushed in Maype mill (Manises, Spain) (Figure 1b) and then it was ground with a ZM 200 centrifugal mill from Retsch (Düsseldorf, Germany) at a speed of 12,000 rpm and finally sieved with a 250 μm mesh filter (Figure 1c). 

### 2.2. Samples Preparation

Bio-DHPE and pinecone powder were dried separately at 60 °C for 48 h in the dehumidifying dryer (MDEO, Industrial Marsé, Barcelona, Spain) in order to remove any residual moisture prior to processing. Two components were mixed prior to being fed into the main hopper of a co-rotating twin-screw extruder (Construcciones Mecanicas Dupra, S.L., Alicante, Spain). This extruder machine has a 25 mm diameter with a length-to-diameter ratio (L/D) of 24. The extrusion process was carried out with a rotating speed of 20 rpm, setting the temperature profile, from the hopper to the die, as follows: 140–145–150–155 °C. The different composites were extruded through a round die to produce strands, which were, pelletized using an air-knife unit. In all cases, residence time was approximately 1 min. In this sense, the Table 1 shows all compositions considered in this work.

The compounded pellets were, thereafter, shaped into standard samples by injection molding in a Meteor 270/75 from Mateu & Solé (Barcelona, Spain). The temperature profile in the injection molding unit was 140 °C (hopper), 150 °C, 155 °C, and 160 °C (injection nozzle). A clamping force of 75 tons was applied while the cavity filling and cooling times were set to 1 and 10 s, respectively. Standard samples for mechanical and thermal characterization with an average thickness of 4 mm were obtained.

### 2.3. Material Characterization

#### 2.3.1. Mechanical Tests 

Tensile tests of injection-molded specimens were carried out in a universal testing machine ELIB 50 from S.A.E. Ibertest (Madrid, Spain) according to ISO 527-1:2012. A 5-kN load cell was used and the cross-head speed was set to 5 mm/min. Shore D hardness was measured with a 676-D durometer from J Bot Instruments (Barcelona, Spain) according to ISO 868:2003. Impact strength was also determined using unnotched pieces with dimensions of 80 × 10 × 4 mm^3^ using Charpy pendulum with an energy of 6 J from Metrotec SA (San Sebastián, Spain) following the guidelines of ISO 179-1:2010. All samples were tested under ambient conditions (23 °C/50% RH), and at least 6 samples of each material were tested, and their values averaged.

#### 2.3.2. Morphology

The morphology of the fracture surfaces of composites from the impact tests, was observed by field emission scanning electron microscopy (FESEM) using a ZEISS ULTRA 55 from Oxford Instruments (Abingdon, UK) microscope. Before placing the samples in the vacuum chamber, the samples were sputtered with a gold-palladium alloy in an EMITECH sputter coating model SC7620 from Quorum Technologies, Ltd. (East Sussex, UK) applying an acceleration voltage of 2 kV. 

#### 2.3.3. Thermal Analysis

Composites samples were analyzed by differential scanning calorimetry (DSC) in a Mettler-Toledo 821 calorimeter (Schwerzenbach, Switzerland). Samples with an average weight between 2 and 3 mg was subjected to three thermal cycles as follows: (1) heating from 20 °C to 160 °C; (2) cooling from 160 °C to 0 °C; (3) heating from 0 °C up to 250 °C. Heating and cooling rates were set to 10 °C/min. All tests were run in nitrogen atmosphere with a flow rate of 66 mL/min using standard sealed aluminum crucibles (40 μL). The degree of crystallinity ($\chi_{c}$) was determined following the equation:

(1) $$\chi_{c}\left(\%\right)=\left[\frac{\Delta H_{m}}{\Delta H_{m}^{0}\cdot \left(1-w\right)}\right]\cdot 100$$

where Δ*H_m_* (J/g) stands for the melting enthalpy of the sample, $\Delta H_{m}^{0}$ (J/g) represents the theoretical melting enthalpy of a fully crystalline BioHDPE, that is, 293.0 J/g [31], and *w* corresponds to the weight fraction of different fibers in the formulation.

Thermogravimetric analysis (TGA) was performed in a LINSEIS TGA 1000 (Selb, Germany). Samples with an average weight between 5 and 7 mg were placed in standard alumina crucibles of 70 µL and subjected to a heating program from 30 °C to 700 °C at a heating rate of 10 °C/min in air atmosphere. The first derivative thermogravimetry (DTG) curves were also determined, expressing the weight loss rate as the function of time.

#### 2.3.4. Thermomechanical Characterization

Thermomechanical properties of composites were obtained by dynamical mechanical thermal analyzer DMA1 from Mettler-Toledo (Schwerzenbach, Switzerland), working in single cantilever flexural conditions. Injection-molded samples with dimensions of 20 × 6 × 2.7 mm^3^ were subjected to a dynamic temperature sweep from −150 °C to 120 °C at a constant heating rate of 2 °C/min. The selected frequency was 1 Hz and the maximum flexural deformation or deflection was set to 10 µm.

#### 2.3.5. Color Measurements 

In order to obtain the color measurements, a Konica CM-3600d Colorflex-DIFF2, from Hunter Associates Laboratory, Inc. (Reston, VA, USA) was used. Color coordinates (L*, a*, b*) were measured according to the following criteria: L* = 0, darkness; L* = 100, lightness; a* represents the green (a* < 0) to red (a* > 0); b* stands for the blue (b* < 0) to yellow (b* > 0) coordinate. The yellowness index for each sample was calculated according to ASTM E313.

#### 2.3.6. Wetting Characterization

Contact angle measurements were carried out with an EasyDrop Standard goniometer model FM140 (KRÜSS GmbH, Hamburg, Deutschland) which is equipped with a video capture kit and analysis software (Drop Shape Analysis SW21; DSA1). Double distilled water was used as test liquid, a drop of it was put in each sample and contact angle measurements were taken at 0, 5, 10, 15, 30, and 45 min after the administration of the water.

#### 2.3.7. Water Uptake Characterization

Injection-molded samples of 4 × 10 × 80 mm^3^ were used. The samples were immersed in distilled water at 23 ± 1 °C. The samples were taken out and weighed weekly using an analytical balance model AG245 from Mettler Toledo Inc. with a precision of ±0.1 mg, after removing the residual water with a dry cloth. The evolution of the water absorption was followed for a period of 15 weeks. All measurements were performed in triplicate. The total absorbed water (Δ*m_t_*) during water immersion was calculated following Equation (2):

(2) $$\Delta m_{t}\left(\%\right)=\left(\frac{W-W_{0}}{W_{0}}\right)\times 100$$

where *W*_0_ is the initial weight and *W*_t_ is the sample weight after an immersion time t of the dry sample before immersion.

## 3. Results

### 3.1. Mechanical Properties

Mechanical characterization of the injection-molded BioHDPE Wood plastic composites with different concentrations of pinecone provides relevant information about the capabilities and possible applications of the developed materials. Table 2 gathers the main mechanical parameters obtained from mechanical tests. 

As it can be seen that the Young Modulus (E) and tensile strength (σ_max_) values of BioHDPE are 818 and 14.6 MPa, respectively. On the other hand, elongation at break (ε_b_) could not be determined due to the value being superior to the elongation capacity of the testing machine. These values are similar to the ones reported by other authors [5]. The incorporation of pinecone produces a significant increase in the stiffness of the material. However, the introduction of PE-g-MA reverts this effect, as it acts as a plasticizer. So, it is necessary to almost duplicate the pinecone content in blends with PE-g-MA in order to reach similar rigidity values. In the case of 20 wt.% pinecone with PE-g-MA, the elastic modulus obtained is 1080 MPa, which is not especially high compared to similar composites with HDPE matrix and lignocellulosic fillers [32]. 

Regarding tensile strength, neat BioHDPE presents the highest value of the studied blends. As the composition of pinecone increases, tensile strength decreases. These effects acutes when PE-g-MA is introduced in the blends. Nonetheless, the reduction in tensile strength does not surpass 30%, this parameter goes from 14.6 MPa for neat BioHDPE, down to 10.6 MPa for 20 wt.% pinecone blend. With regard to elongation at break, the incorporation of loads provokes a great reduction in the elongation of the material, while the compatibilizer increases this parameter. This fact allows the material to preserve enough ductility for a wide range of applications. This trend is corroborated by impact strength results.

BioHDPE shows the greatest impact strength values. The addition of pinecone increases the fragility of the material, but the presence of the compatibilizer in the blend improves this parameter considerably from 1.5 (kJ/m^2^) for 5 wt.% content in pinecone without compatibilizer to 1.7 (kJ/m^2^) for the same amount of pinecone with PE-g-MA. This behavior highlights the plasticizing effect that PE-g-MA exerts over the composite, increasing its ductile properties but slightly reducing its tensile strength. Higher composition of pinecone produces an increase in the fragility of the composites, reaching values near 1.3 (kJ/m^2^) for a 20 wt.% content in pinecone. This behavior has also been reported in other composites with HDPE and lignocellulosic fillers [33].

With respect to hardness, results show that PE-g-MA slightly increases Shore D hardness. As it can be seen, BioHDPE has a Shore D hardness of 62.3, similar to that of BioHDPE/5PC (62.7). On the contrary, once the compatibilizer is added, the greater the pinecone content in the blend the higher the hardness, reaching a maximum value of 65.4 for 20 wt.% of pinecone. It should be remarked that the variability in hardness results is quite low and in some cases it lacks representativeness.

### 3.2. Morphology of BioHDPE-Pinecone Composites

The internal structure of the composites is often closely related to their mechanical performance. Especially the interaction between the filler particle surface and the surface of the polymer matrix. Another important point is the size and shape of the filler particles.

**Figure 2:**
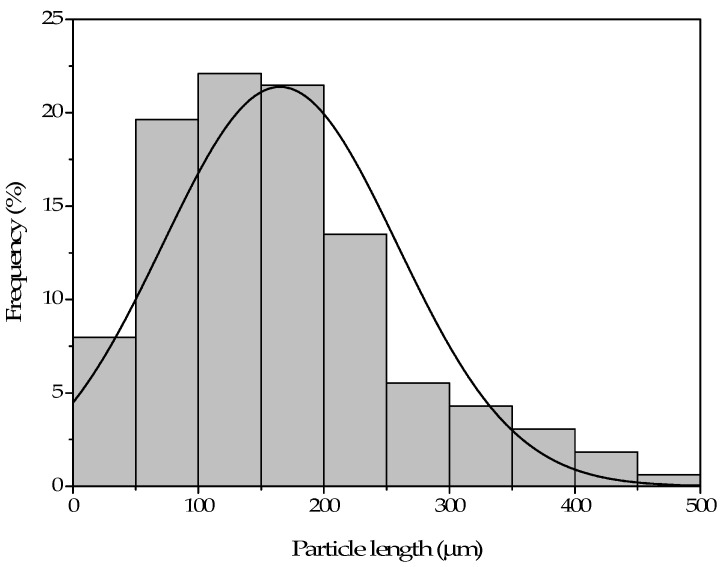
Histogram of the pinecone particles length.

Figure 2 shows the length histogram for the pinecone particles, obtained from FESEM images. As it can be observed, the average size of the pinecone particles is approximately 150 μm, being 66% of the particles between 50 and 200 μm. Particle size can negatively influence mechanical properties when the particles are too large [34].

**Figure 3:**
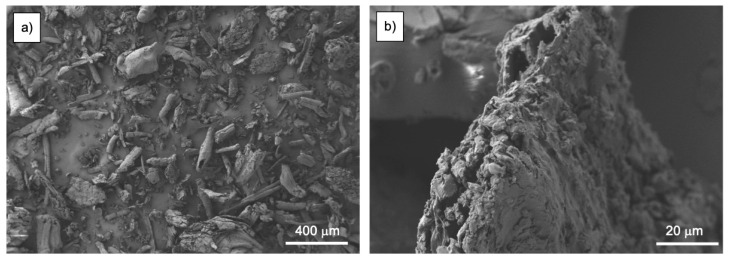
Field emission scanning electron microscopy (FESEM) images of pinecone particles taken at: (**a**) 50× with a scale marker of 400 μm; (**b**) 1000× with a scale marker of 40 μm.

Regarding pinecone particles morphology, Figure 3 shows the FESEM images at different magnifications. 

The shape of the particles is irregular, with a commonly observed cylindrical geometry. At higher magnification, great levels of roughness can be appreciated, which will positively influence the filler–matrix interaction, improving the mechanical performance of the material. The roughness observed is typical in pinecone particles, and it is a consequence of the crushing process, which breaks particles into tiny compact and porous particles with great roughness. This phenomenon also occurs in other similar lignocellulosic compounds [35].

### 3.3. Morphology of BioHDPE-Pinecone Composites

**Figure 4:**
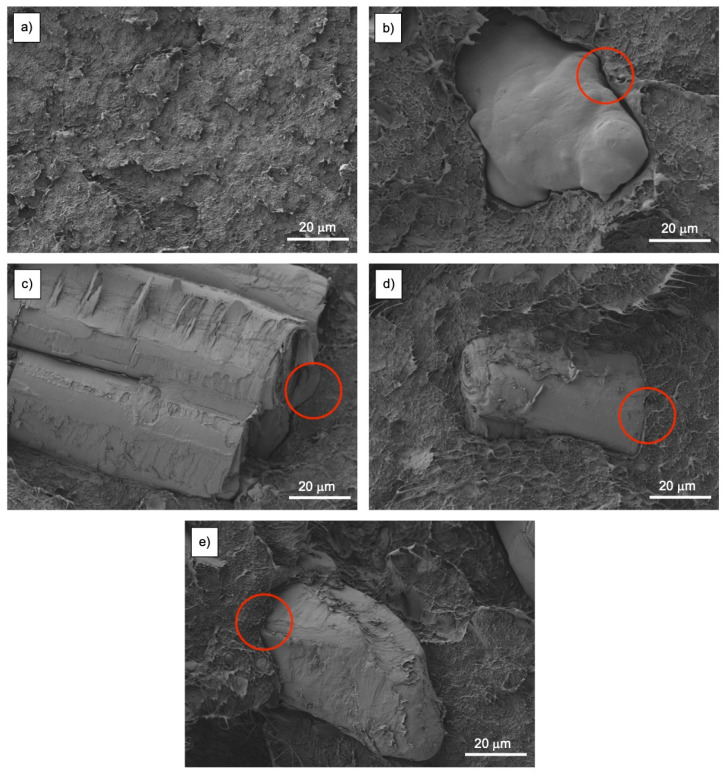
Field emission scanning electron microscopy (FESEM) images at 1000× of the impact fractured surfaces of: (**a**) BioHDPE; (**b**) BioHDPE/5PC; (**c**) BioHDPE/5PC/PE-g-MA; (**d**) BioHDPE/10PC/PE-g-MA; (**e**) BioHDPE/20PC/PE-g-MA.

The interaction in the interface between pinecone particles and BioHDPE matrix defines the mechanical properties of the composite. Figure 4 shows the FESEM images of the surface of fractured impact test samples of different BioHDPE/pinecone composites with different pinecone contents and PE-g-MA. Figure 4a shows the morphology of BioHDPE. It is the typical surface of a ductile polymer, irregular, rough, and cavernous, as it has been reported in other works [5]. 

Figure 4b shows the surface of the BioHDPE/5PC sample. The great void (red circle) that is observed between the pinecone particle and the BioHDPE matrix is evidence of the lack of cohesion, which translates in poor mechanical properties, especially in terms of elongation at break. The incorporation of PE-g-MA as a compatibilizer shows a clear reduction in the gap between the pinecone particle and the matrix that envelops it, as it can be seen in Figure 4c. This increment in cohesion is strictly connected with an improvement in the ductile mechanical properties of the composite with compatibilizer in comparison with the composite without it. This effect can also be observed in the blends with 10 and 20 wt.% of pinecone, as it is portraited in Figure 4d and Figure 4e, respectively.

### 3.4. Thermal Properties of BioHDPE-Pinecone Composites

**Figure 5:**
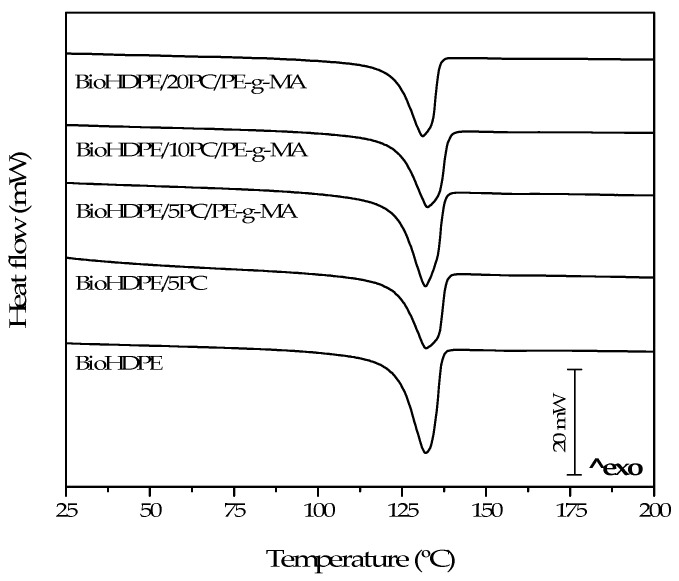
Differential scanning calorimetry (DSC) thermograms of BioHDPE-pinecone composites.

Figure 5 shows the DSC thermograms obtained during the second heating cycle of the BioHDPE/PC composites. On the other hand, Table 3 sums up the main data acquired during thermal analysis. With regard to the thermograms, the obtained curves only show the melting temperature of the BioHDPE composites. Glass transition temperature cannot be observed because it is located at approximately −100 °C. BioHDPE presents a melting peak (T_m_) at 131.0 °C with a crystallinity value of X_c_ 61.9%. These are similar values to those obtained by other authors [36]. 

The studied composites did not show significant differences in terms of the melting point, obtaining values very close to 131 °C. Regarding melting enthalpy, important changes appear depending on the pinecone composition and the use of PE-g-MA as a compatibilizer. As a result, crystallinity percentage also changes drastically. In this sense, the addition of 5 wt.% pinecone reduces the nucleation process due to the particle–particle contact, as the available space for crystal growth becomes limited [36,37,38]. As a result, crystallinity reduces from 61.9% for BioHDPE, to 57.2% for BioHDPE/5PC. On the contrary, when PE-g-MA is introduced, the dispersion of the pinecone particles in the polymer matrix improves, contributing to reduce polymer–polymer interactions, thus, supporting crystal formation [39,40]. For the 10 wt.% pinecone blend, the compatibilizing effect of PE-g-MA that increases crystallinity gets compensated by the contrary effect exerted by the higher composition of pinecone, obtaining an inferior value of 58.1%. In the case of 20 wt.% pinecone sample, the effect of the filler increases and crystallinity value goes down to 57.6%.

**Figure 6:**
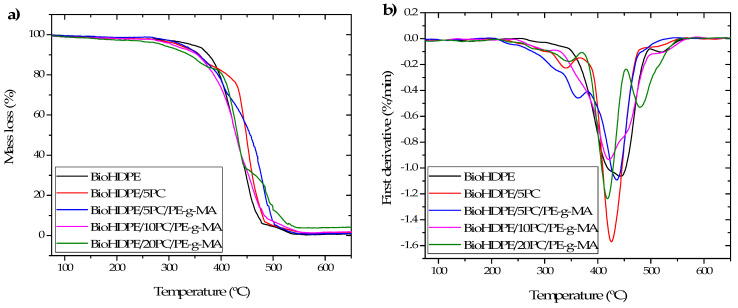
TGA curves of BioHDPE-pinecone composites: (**a**) thermogravimetric analysis (TGA) curves; (**b**) first derivative (DTG).

With regard to thermal stability of the studied composites, Figure 6 shows the thermogravimetric curves and their first derivative (DTG). Moreover, Table 4 gathers the main degradation parameters for all the blends. Neat BioHDPE shows a degradation temperature for a mass loss of 5% (T_5%_) of 343 °C, a degradation temperature (T_deg_) of 443 °C, and a residual mass at 700 °C of 0.9%. These values are typical of this kind of material and have also been reported in other works [41]. The addition of lignocellulosic fillers provokes a reduction in the thermal stability of the composite, resulting in a decrease both in T_5%_ and T_deg_. The incorporation of 5 wt.% pinecone results in a reduction down to 317 °C and 451 °C, respectively. Otherwise, the addition of the PE-g-MA compatibilizer provides thermal stability to the composite, due to its ability to reticulate polymer chains altogether with an improvement in the dispersion of the particles, which triggers a delay in the maximum degradation peak [33]. It is for this reason that, for the same quantity of pinecone with PE-g-MA, the values of T_5%_ and T_deg_ suffer an increase of 8 and 11 °C, respectively. Once the percentage of filler is augmented, the degradation of the composite becomes faster, obtaining significantly lower values of 306 and 421 °C for T_5%_ and T_deg_, respectively, for the BioHDPE/10PC/PE-g-MA composite. In the case of the 20 wt.% pinecone composite, the values of T_5%_ and T_deg_ decrease down to 287 and 416 °C, respectively. This implies a reduction of the 16 and 6% in relation to neat BioHDPE.

It should be remarked that in the DTG curves of the composite containing pinecone, the degradation of the different compounds present in the lignocellulosic particles becomes clearly visible: hemicellulose degradation at 280–340 °C, cellulose degradation at 340–450 °C, and lignin degradation at 450–509 °C [42,43]. 

With respect to residual mass results, the introduction of lignocellulosic particles provokes an increase in residual mass due to the fact that these particles do not degrade completely at 700 °C [44]. The residual mass values increase from 1.2% for the compound with 5 wt.% of pinecone up to 4.3% for the 20 wt.% pinecone composite.

### 3.5. Thermomechanical Properties of BioHDPE-Pinecone Composites

**Figure 7:**
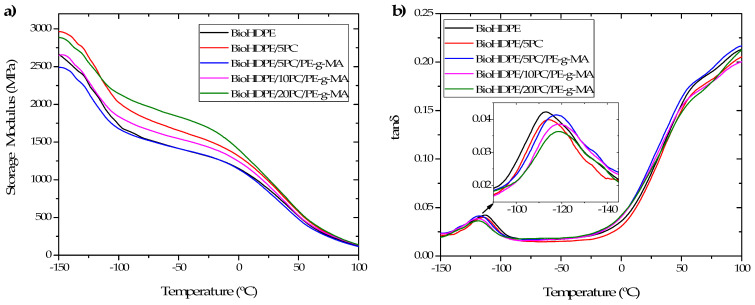
DMTA curves of BioHDPE-pinecone composites: (**a**) Storage modulus (G′) and (**b**) dynamic damping factor (tan δ).

Figure 7 shows the thermodynamic curves obtained by means of DMTA for all the BioHDPE/pinecone blends. Figure 7a shows the evolution of the storage modulus (G′) in the temperature range −150–100 °C. As it was expected, a reduction of G′ is observed from values in the range 2500–3000 MPa at −150 °C down to values near 120 MPa at 100 °C. Table 5 gathers G′ values at different temperatures (−150 °C; 0 °C and 75 °C) and the values corresponding to the dynamic damping factor (tan δ), which is indicative of the T_g_ of the BioHDPE matrix in the blends. 

Regarding BioHDPE, at −150 °C an initial value of G′ of 2658 MPa is obtained. Then, a rapid decrease in the storage modulus is observed, related with the glass transition of the material, until −100 °C approximately. The next sudden decrease (at 0 °C) is related to the softening of the polymer matrix [5]. With regard to the composites, the incorporation of 5 wt.% pinecone into the matrix provokes an initial increase of G′ up to 2965 MPa. The addition of PE-g-MA compatibilizer tends to decrease G′, countering the increasing effect exerted by the pinecone filler. This effect is seen in the compatibilized sample, which at −150 °C presents a G′ value of 2490 MPa, a lower value than neat BioHDPE. Once the percentage of pinecone is increased, G′ augments up to 2658 and 2886 MPa for 10 and 20 wt.% pinecone samples, respectively. This tendency is similar to that found at higher temperatures, although the 5 wt.% pinecone non-compatibilized sample suffers a greater fall in G′ than the samples with high content in pinecone. Thus, the 20 wt.% pinecone sample is the one with the higher storage modulus at 0 and 75 °C (1409 and 299 MPa, respectively).

Figure 7b shows the evolution of the dynamic damping factor (tan δ) with temperature. Neat BioHDPE presents a peak at 112.8 °C, corresponding to the γ relaxation and related to its glass transition temperature [45]. A second relaxation is observed, called α-relaxation between 50 and 120 °C, related to an interlaminar shearing process that occurs during heating. α relaxation can be separated in two subprocesses (α y α′) due to the heterogeneity of the crystalline regions in the material [46]. The addition of pinecone provokes a slight increase, which results insignificant. However, the incorporation of PE-g-MA does produce a greater change of up to 5 °C. Higher proportions of pinecone increase T_g_ because pinecone particles immobilize BioHDPE polymeric chains.

### 3.6. Colour Measurement and Visual Appearance 

**Figure 8:**
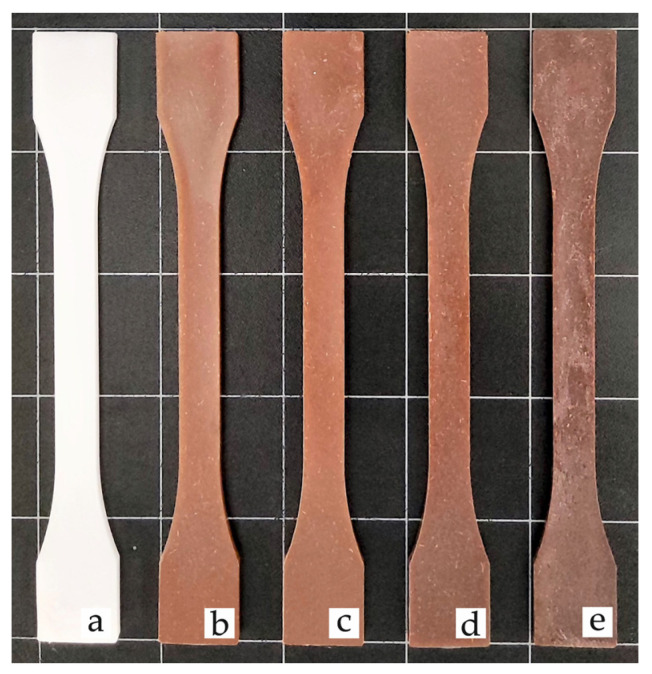
Visual appearance of injection-molded pieces of BioHDPE composites: (**a**) BioHDPE; (**b**) BioHDPE/5PC; (**c**) BioHDPE/5PC/PE-g-MA; (**d**) BioHDPE/10PC/PE-g-MA; (**e**) BioHDPE/20PC/PE-g-MA.

One of the main objectives when developing WPC is obtaining a wood-like visual appearance. For this reason, colorimetric analysis has been carried out in this work. Table 6 gathers the values of the color coordinates L*, a*, b* of the BioHDPE compounds with pinecone, while Figure 8 shows the visual appearance of tensile test samples. All samples are opaque, mainly due to the semicrystalline nature of BioHDPE [47].

BioHDPE shows a great luminance L* as a result of its characteristic white color, in contrast with the intense brown color of the pinecone composites, which provokes an important reduction in their luminance. Interestingly, as the pinecone content increases, the sample acquires darker shades, as it can be inferred from the decrease in luminance (4 units) from 5 wt.% pinecone sample to 20 wt.% pinecone sample. With regard to color coordinate a*, a great change in the samples can be appreciated, going from negative values (green) to positive values (red) in the pinecone composites [48]. In terms of color coordinate b*, which defines blue (negative) or yellow (positive) color, negative values for BioHDPE are observed (−3.12), while pinecone composites exhibit positive values as a result of their darkening and characteristic brown color. 

The visual appearance shown by pinecone composites perfectly matches the definition of wood plastic composites, as they present brown colors very similar to those of natural woods, which gives them a wide range of applications.

### 3.7. Water Uptake Characterization

**Figure 9:**
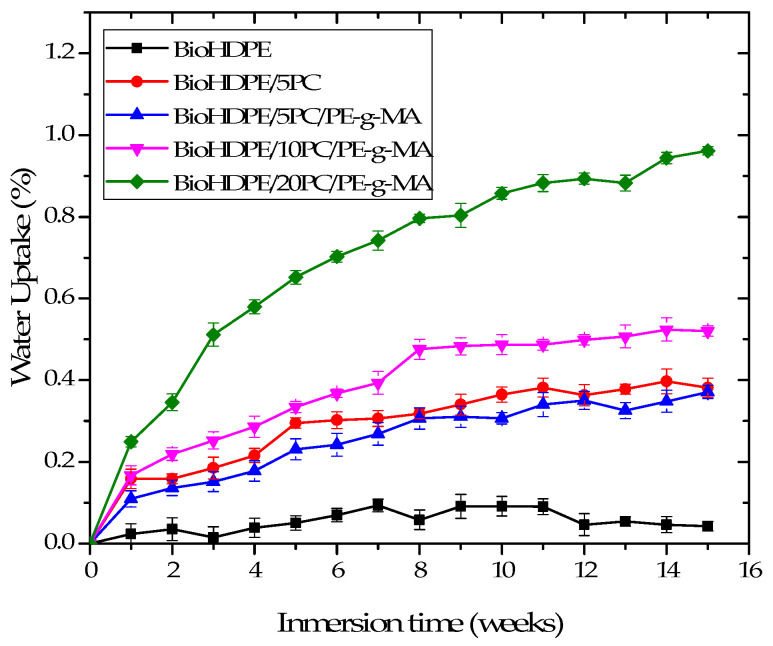
Water uptake of the injection-molded pieces made of BioHDPE-pinecone composites.

One of the main drawbacks of composites with polymeric matrices and lignocellulosic fillers such as pinecone is their great hydrophilic nature, which limits their use in certain industries and applications, as they become strong water-absorbents. Figure 9 shows the water absorption evolution of BioHDPE composites with pinecone, after a period of 15 weeks of distilled water immersion. 

As it was expected, neat BioHDPE hardly absorbed any water in all the immersion time, with an asymptotic value of 0.03%, confirming the hydrophobic behavior of this material [49]. As it can be observed, the introduction of 5 wt.% pinecone provokes a strong increase in water absorption, reaching 0.39% with respect to the initial weight of the sample at 15 weeks. This is mainly ascribed to the presence of hydroxyl groups present in the lignocellulosic particles of pinecone, which enhances moisture absorption [50]. This is the reason why higher concentrations of pinecone induce higher levels of water absorption, reaching values of 0.52% and 0.96% for the 10 and 20 wt.% pinecone samples, respectively. The inclusion of PE-g-MA in the blends slightly reduces water uptake, which could be related to the fact that a greater interaction between BioHDPE and PC somehow blocks moisture absorption.

### 3.8. Wetting Properties

**Figure 10:**
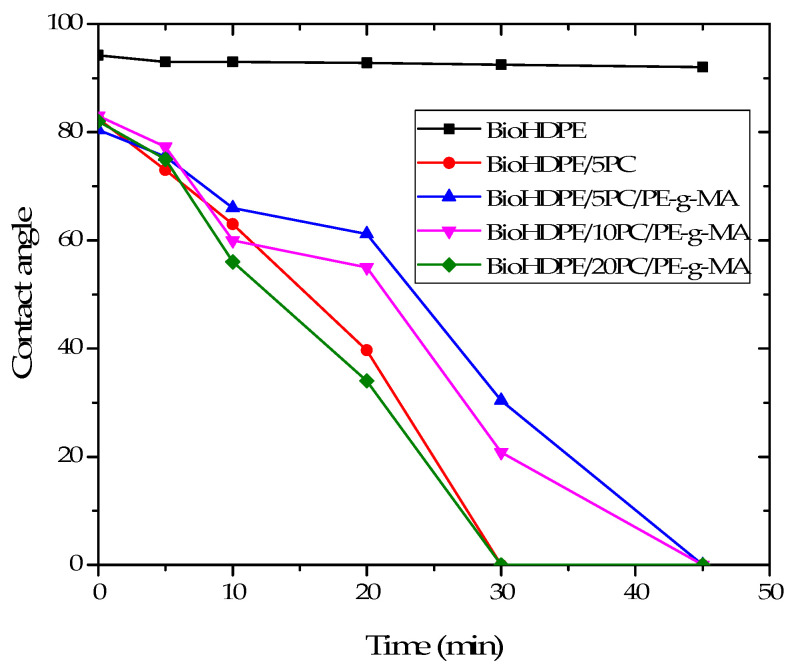
Water contact angle of different Bio-HDPE-pinecone composites at several times of exposure.

With the objective of evaluating the behavior of the pinecone composites against water, the contact angle at different times after applying one drop of distilled water in the surface of each one of the samples was measured. A high contact angle value is indicative of a poor affinity for water (hydrophobicity), while a low contact angle is related to a strong hydrophilic behavior. Figure 10 shows the variation of water contact angles with time for all the BioHDPE/pinecone composites. As it can be seen, initially (0 min exposure time) all the samples present values superior to 65°, which makes them hydrophobic according to the hydrophilic threshold established by Vogler [51]. This is ascribed to the non-polar nature of BioHDPE, which is formed only by C-H bonds. These bonds have practically no difference in electronegativity. This is also the reason why BioHDPE shows a constant contact angle of 94° for all the exposure times. When pinecone particles are introduced, a considerable reduction of about 13% in the contact angle is observed. This could be ascribed to cellulose, hemicellulose, and lignin, which are present in pinecone particles (hydroxyl and carbonyl groups). These groups provide polarity to the material and can form hydrogen bonds with water (polar solvent), giving higher wettability [52]. Furthermore, as it can be observed, contact angle considerably reduces over time. The more intense phenomenon is the higher the pinecone proportion, although the presence of PE-g-MA reduces this effect. Thereby, it can be observed how the sample with 5 wt.% of pinecone with PE-g-MA exhibits a contact angle value of 30.4° after 30 min of exposure time, while in the non-compatibilized sample with the same pinecone proportion the water drop completely disappeared. This fact is linked to a greater interaction between the filler and the matrix, which avoids a higher water absorption. Higher pinecone compositions make the water drop to completely disappear after 45 min of exposure time, showing that the compatibilizing effect is not enough in this case to counter the hydrophilicity of the lignocellulosic pinecone particles.

The results presented here are consistent with the ones obtained in the water uptake test, concluding that BioHDPE composites obtain more affinity for water as the pinecone content increases in the blends, although the use of PE-g-MA tends to slightly reduce this behavior.

## 4. Conclusions and Future Perspectives

The results obtained in this work allow to validate the incorporation of pinecone particles for the obtention of WPCs with a relatively low cost. The use of highly abundant reinforcing fillers and a very low cost such as pinecone offer can also increase the performance of the obtained material. It has been demonstrated that it is possible to obtain good mechanical response from pinecone composites up to 20 wt.% of pinecone. The use of PE-g-MA as a compatibilizer has effectively increased the ductile mechanical properties of the composites. Thermal stability also improves considerably with the use of PE-g-MA, even at high proportions of pinecone. Moreover, the intrinsic great water absorption of the composites with lignocellulosic particles is lowered by effect of the compatibilizer, improving the behavior against water, which allows the use of these materials even in some outdoor environments. Regarding visual appearance, characteristic brown colors similar to those of some natural woods have been obtained with the addition of pinecone. It has also been demonstrated that the affinity between the non-polar polymeric matrix and the lignocellulosic particles allows a general upgrade of the capabilities of the materials. Moreover, compositions of up to 20 wt.% of pinecone have been used, which drastically reduces the cost of the final product. All in all, it has been possible to develop a completely sustainable material with potential application in gardening furniture, owing to its wood-like color and its good outdoor performance. In short, the results obtained in this work allow validating the wood plastic composites obtained from a very abundant natural resource such as pinecone, the use of compatibilizers has a great impact on the performance, allowing the addition of a higher percentage of reinforcement without reducing the performance range. The development of new compatibilizers more suitable for this composite is foreseeably one of the objects of study for future work related to this field.

## Figures and Tables

**Table 1:** Summary of compositions according to the weight content (wt%) of Bio-HDPE (BioHDPE); pinecone (PC) powder and polyethylene-grafted maleic anhydride (PE-g-MA).

Code	BioHDPE (wt.%)	Pinecone (wt.%)	PE-g-MA (phr)
BioHDPE	100	0	0
BioHDPE/5PC	95	5	0
BioHDPE/5PC/PE-g-MA	95	5	2
BioHDPE/10PC/PE-g-MA	90	10	2
BioHDPE/20PC/PE-g-MA	80	20	2

**Table 2:** Summary of mechanical properties of the injection-molded parts of BioHDPE composites in terms of: tensile modulus (E), maximum tensile strength (σ_max_), and elongation at break (ε_b_), Shore D hardness, and impact strength.

Code	E (MPa)	σ_max_ (MPa)	ε_b_ (%)	Shore D Hardness	Impact Strength (kJ/m^2^)
BioHDPE	818 ± 12	14.6 ± 0.7	NB *	62.3 ± 0.9	2.0 ± 0.2
BioHDPE/5PC	920 ± 16	13.9 ± 0.6	45.1± 2.3	62.7 ± 0.5	1.5 ± 0.1
BioHDPE/5PC/PE-g-MA	840 ± 15	13.6 ± 0.9	50.1 ± 4.9	64.1 ± 0.5	1.7 ± 0.2
BioHDPE/10PC/PE-g-MA	890 ± 14	11.7 ± 0.5	23.5 ± 1.2	64.5 ± 0.7	1.4 ± 0.1
BioHDPE/20PC/PE-g-MA	1080 ± 25	10.6 ± 0.6	15.6 ± 0.4	65.4 ± 0.8	1.3 ± 0.1

* NB → No Break.

**Table 3:** Main thermal parameters of the of BioHDPE-pinecone composites in terms of: melting temperature (T_m_), normalized melting enthalpy (ΔH_m_), and percentage of crystallinity (χ_c_).

Code	T_m_ (°C)	ΔH_m_ (J/g)	χ_c_ (%)
BioHDPE	131.0 ± 1.1	181.4 ± 1.3	61.9 ± 1.1
BioHDPE/5PC	131.3 ± 1.0	159.1 ± 1.0	57.2 ± 0.9
BioHDPE/5PC/PE-g-MA	130.8 ± 0.9	175.6 ± 1.2	63.1 ± 0.9
BioHDPE/10PC/PE-g-MA	131.7 ± 0.8	153.3 ± 0.9	58.1 ± 0.8
BioHDPE/20PC/PE-g-MA	130.3 ± 0.7	135.1 ± 1.1	57.6 ± 0.7

**Table 4:** Main thermal degradation parameters of the samples of BioHDPE/PC composites in terms of: temperature at mass loss of 5% (T_5%_), maximum degradation rate (peak) temperature (T_deg_), and residual weight at 700 °C.

Code	T_5%_ (°C)	T_deg_ (°C)	Residual Mass (%)
BioHDPE	343 ± 1.5	443 ± 1.6	0.9 ± 0.1
BioHDPE/5PC	317 ± 1.2	425 ± 1.3	1.2 ± 0.2
BioHDPE/5PC/PE-g-MA	325 ± 1.1	436 ± 1.6	1.2 ± 0.3
BioHDPE/10PC/PE-g-MA	306 ± 1.1	421 ± 1.9	2.1 ± 0.4
BioHDPE/20PC/PE-g-MA	287 ± 0.9	416 ± 1.1	4.3 ± 0.3

**Table 5:** Main thermomechanical parameters of the injection-molded parts of Bio-HDPE with different fibers.

Code	G′ (MPa) at −150 °C	G′ (MPa) at 0 °C	G′ (MPa) at 75 °C	T_g_ (°C)
BioHDPE	2658 ± 12	1154 ± 15	259 ± 8	−112.8 ± 0.8
BioHDPE/5PC	2965 ± 14	1315 ± 19	288 ± 6	−113.0 ± 0.9
BioHDPE/5PC/PE-g-MA	2490 ± 21	1145 ± 12	244 ± 7	−117.2 ± 0.9
BioHDPE/10PC/PE-g-MA	2658 ± 26	1245 ± 21	274 ± 8	−119.0 ± 1.4
BioHDPE/20PC/PE-g-MA	2886 ± 19	1409 ± 16	299 ± 9	−118.8 ± 1.2

**Table 6:** Color indexes (L*, a*, b*) of injection-molded pieces of BioHDPE composites.

Code	L*	a*	b*
BioHDPE	74.82 ± 0.22	−2.52 ± 0.05	−3.12 ± 0.22
BioHDPE/5PC	36.98 ± 0.05	8.18 ± 0.08	10.0 ± 0.12
BioHDPE/5PC/PE-g-MA	36.49 ± 0.13	8.02 ± 0.09	9.41 ± 0.14
BioHDPE/10PC/PE-g-MA	34.07 ± 0.12	6.18 ± 0.11	6.41± 0.11
BioHDPE/20PC/PE-g-MA	32.34 ± 0.19	5.87 ± 0.10	6.08 ± 0.20

## Data Availability

Not applicable.

## References

[1] Yaqoob A.A., Ibrahim M.N.M., Ahmad A., Reddy A.V.B. (2021). Toxicology and environmental application of carbon nanocomposite. Environmental Remediation through Carbon Based Nano Composites.

[2] Liminana P., Garcia-Sanoguera D., Quiles-Carrillo L., Balart R., Montanes N. (2018). Development and characterization of environmentally friendly composites from poly (butylene succinate) (PBS) and almond shell flour with different compatibilizers. Compos. Part B Eng..

[3] Babu R.P., O’connor K., Seeram R. (2013). Current progress on bio-based polymers and their future trends. Prog. Biomater..

[4] Önal E., Uzun B.B., Pütün A.E. (2014). Bio-oil production via co-pyrolysis of almond shell as biomass and high density polyethylene. Energy Convers. Manag..

[5] Quiles-Carrillo L., Montanes N., Jorda-Vilaplana A., Balart R., Torres-Giner S. (2019). A comparative study on the effect of different reactive compatibilizers on injection-molded pieces of bio-based high-density polyethylene/polylactide blends. J. Appl. Polym. Sci..

[6] Torres-Giner S., Torres A., Ferrándiz M., Fombuena V., Balart R. (2017). Antimicrobial activity of metal cation-exchanged zeolites and their evaluation on injection-molded pieces of bio-based high-density polyethylene. J. Food Saf..

[7] Vasile C., Râpă M., Ştefan M., Stan M., Macavei S., Darie-Niţă R., Barbu-Tudoran L., Vodnar D., Popa E., Ştefan R. (2017). New PLA/ZnO: Cu/Ag bionanocomposites for food packaging. Express Polym. Lett..

[8] Umar K., Yaqoob A., Ibrahim M., Parveen T., Safian M. (2020). Environmental applications of smart polymer composites. Smart Polym. Nanocompos. Biomed. Environ. Appl..

[9] Dahy H. (2017). Biocomposite materials based on annual natural fibres and biopolymers–Design, fabrication and customized applications in architecture. Constr. Build. Mater..

[10] Sinha A.K., Narang H.K., Bhattacharya S. (2017). Mechanical properties of natural fibre polymer composites. J. Polym. Eng..

[11] Agüero Á., Garcia-Sanoguera D., Lascano D., Rojas-Lema S., Ivorra-Martinez J., Fenollar O., Torres-Giner S. (2020). Evaluation of different compatibilization strategies to improve the performance of injection-molded green composite pieces made of polylactide reinforced with short flaxseed fibers. Polymers.

[12] Delgado-Aguilar M., Julián F., Tarrés Q., Méndez J., Mutjé P., Espinach F. (2017). Bio composite from bleached pine fibers reinforced polylactic acid as a replacement of glass fiber reinforced polypropylene, macro and micro-mechanics of the Young’s modulus. Compos. Part B Eng..

[13] Garcia-Garcia D., Quiles-Carrillo L., Montanes N., Fombuena V., Balart R. (2018). Manufacturing and Characterization of Composite Fibreboards with Posidonia oceanica Wastes with an Environmentally-Friendly Binder from Epoxy Resin. Materials.

[14] Carbonell-Verdu A., Boronat T., Quiles-Carrillo L., Fenollar O., Dominici F., Torre L. (2020). Valorization of Cotton Industry Byproducts in Green Composites with Polylactide. J. Polym. Environ..

[15] Väisänen T., Haapala A., Lappalainen R., Tomppo L. (2016). Utilization of agricultural and forest industry waste and residues in natural fiber-polymer composites: A review. Waste Manag..

[16] Lieder M., Rashid A. (2016). Towards circular economy implementation: A comprehensive review in context of manufacturing industry. J. Clean. Prod..

[17] Bilitewski B. (2012). The circular economy and its risks. Waste Manag..

[18] Corrado A., Polini W. (2019). Measurement of high flexibility components in composite material by touch probe and force sensing resistors. J. Manuf. Process..

[19] Chen J., Gao X. (2019). Directional dependence of electrical and thermal properties in graphene-nanoplatelet-based composite materials. Results Phys..

[20] Harada J., de Souza A.G., de Macedo J.R., Rosa D.S. (2019). Soil culture: Influence of different natural fillers incorporated in biodegradable mulching film. J. Mol. Liq..

[21] Omrani E., Menezes P.L., Rohatgi P.K. (2016). State of the art on tribological behavior of polymer matrix composites reinforced with natural fibers in the green materials world. Eng. Sci. Technol. Int. J..

[22] Assi A., Bilo F., Zanoletti A., Ducoli S., Ramorino G., Gobetti A., Zacco A., Federici S., Depero L.E., Bontempi E. (2020). A Circular Economy Virtuous Example—Use of a Stabilized Waste Material Instead of Calcite to Produce Sustainable Composites. Appl. Sci..

[23] Quiles-Carrillo L., Montanes N., Lagaron J.M., Balart R., Torres-Giner S. (2018). On the use of acrylated epoxidized soybean oil as a reactive compatibilizer in injection-molded compostable pieces consisting of polylactide filled with orange peel flour. Polym. Int..

[24] Hoffmann R., Morais D., Braz C., Haag K., Wellen R., Canedo E., de Carvalho L., Koschek K. (2019). Impact of the natural filler babassu on the processing and properties of PBAT/PHB films. Compos. Part A Appl. Sci. Manuf..

[25] Quiles-Carrillo L., Montanes N., Sammon C., Balart R., Torres-Giner S. (2018). Compatibilization of highly sustainable polylactide/almond shell flour composites by reactive extrusion with maleinized linseed oil. Ind. Crop. Prod..

[26] Liu W., Misra M., Askeland P., Drzal L.T., Mohanty A.K. (2005). ‘Green’composites from soy based plastic and pineapple leaf fiber: Fabrication and properties evaluation. Polymer.

[27] Kapatel P.M. (2019). Investigation of green composite: Preparation and characterization of alkali-treated jute fabric-reinforced polymer matrix composites. J. Nat. Fibers.

[28] Srivastava K.R., Singh M.K., Mishra P.K., Srivastava P. (2019). Pretreatment of banana pseudostem fibre for green composite packaging film preparation with polyvinyl alcohol. J. Polym. Res..

[29] Torres-Giner S., Hilliou L., Melendez-Rodriguez B., Figueroa-Lopez K.J., Madalena D., Cabedo L., Covas J., Vicente A.A., Lagaron J. (2018). Melt processability, characterization, and antibacterial activity of compression-molded green composite sheets made of poly (3-hydroxybutyrate-co-3-hydroxyvalerate) reinforced with coconut fibers impregnated with oregano essential oil. Food Packag. Shelf Life.

[30] Agüero Á., Lascano D., Garcia-Sanoguera D., Fenollar O., Torres-Giner S. (2020). Valorization of linen processing by-products for the development of injection-molded green composite pieces of polylactide with improved performance. Sustainability.

[31] Quiles-Carrillo L., Montanes N., Fombuena V., Balart R., Torres-Giner S. (2020). Enhancement of the processing window and performance of polyamide 1010/bio-based high-density polyethylene blends by melt mixing with natural additives. Polym. Int..

[32] Jorda-Reolid M., Gomez-Caturla J., Ivorra-Martinez J., Stefani P.M., Rojas-Lema S., Quiles-Carrillo L. (2021). Upgrading Argan Shell Wastes in Wood Plastic Composites with Biobased Polyethylene Matrix and Different Compatibilizers. Polymers.

[33] Singh V.P., Vimal K., Kapur G., Sharma S., Choudhary V. (2016). High-density polyethylene/halloysite nanocomposites: Morphology and rheological behaviour under extensional and shear flow. J. Polym. Res..

[34] Crespo J.E., Balart R., Sanchez L., Lopez J. (2007). Mechanical behaviour of vinyl plastisols with cellulosic fillers. Analysis of the interface between particles and matrices. Int. J. Adhes. Adhes..

[35] Crespo J.E., Sanchez L., Parres F., Lopez J. (2007). Mechanical and morphological characterization of PVC plastisol composites with almond husk fillers. Polym. Compos..

[36] Essabir H., Bensalah M.O., Rodrigue D., Bouhfid R., Qaiss A.e.K. (2016). Biocomposites based on Argan nut shell and a polymer matrix: Effect of filler content and coupling agent. Carbohydr. Polym..

[37] Quiles-Carrillo L., Montava-Jorda S., Boronat T., Sammon C., Balart R., Torres-Giner S. (2020). On the Use of Gallic Acid as a Potential Natural Antioxidant and Ultraviolet Light Stabilizer in Cast-Extruded Bio-Based High-Density Polyethylene Films. Polymers.

[38] Tas C.E., Hendessi S., Baysal M., Unal S., Cebeci F.C., Menceloglu Y.Z., Unal H. (2017). Halloysite Nanotubes/Polyethylene Nanocomposites for Active Food Packaging Materials with Ethylene Scavenging and Gas Barrier Properties. Food Bioprocess Technol..

[39] Chieng B.W., Ibrahim N.A., Then Y.Y., Loo Y.Y. (2014). Epoxidized Vegetable Oils Plasticized Poly(lactic acid) Biocomposites: Mechanical, Thermal and Morphology Properties. Molecules.

[40] Liu M., Guo B., Du M., Chen F., Jia D. (2009). Halloysite nanotubes as a novel beta-nucleating agent for isotactic polypropylene. Polymer.

[41] Montanes N., Garcia-Sanoguera D., Segui V.J., Fenollar O., Boronat T. (2018). Processing and Characterization of Environmentally Friendly Composites from Biobased Polyethylene and Natural Fillers from Thyme Herbs. J. Polym. Environ..

[42] Essabir H., Hilali E., Elgharad A., El Minor H., Imad A., Elamraoui A., Al Gaoudi O. (2013). Mechanical and thermal properties of bio-composites based on polypropylene reinforced with Nut-shells of Argan particles. Mater. Des..

[43] Ouajai S., Shanks R.A. (2005). Composition, structure and thermal degradation of hemp cellulose after chemical treatments. Polym. Degrad. Stab..

[44] Liminana P., Quiles-Carrillo L., Boronat T., Balart R., Montanes N. (2018). The Effect of Varying Almond Shell Flour (ASF) Loading in Composites with Poly(Butylene Succinate (PBS) Matrix Compatibilized with Maleinized Linseed Oil (MLO). Materials.

[45] Castro D.O., Ruvolo-Filho A., Frollini E. (2012). Materials prepared from biopolyethylene and curaua fibers: Composites from biomass. Polym. Test..

[46] Pegoretti A., Ashkar M., Migliaresi C., Marom G. (2000). Relaxation processes in polyethylene fibre-reinforced polyethylene composites. Compos. Sci. Technol..

[47] Shen L., Nickmans K., Severn J., Bastiaansen C.W.M. (2016). Improving the Transparency of Ultra-Drawn Melt-Crystallized Polyethylenes: Toward High-Modulus/High-Strength Window Application. Acs Appl. Mater. Interfaces.

[48] Quiles-Carrillo L., Fenollar O., Balart R., Torres-Giner S., Rallini M., Dominici F., Torre L. (2020). A comparative study on the reactive compatibilization of melt-processed polyamide 1010/polylactide blends by multi-functionalized additives derived from linseed oil and petroleum. Express Polym. Lett..

[49] Kuciel S., Jakubowska P., Kuzniar P. (2014). A study on the mechanical properties and the influence of water uptake and temperature on biocomposites based on polyethylene from renewable sources. Compos. Part B Eng..

[50] Quiles-Carrillo L., Montanes N., Garcia-Garcia D., Carbonell-Verdu A., Balart R., Torres-Giner S. (2018). Effect of different compatibilizers on injection-molded green composite pieces based on polylactide filled with almond shell flour. Compos. Part B Eng..

[51] Vogler E.A. (1998). Structure and reactivity of water at biomaterial surfaces. Adv. Colloid Interface Sci..

[52] Lee J.H., Park J.W., Lee H.B. (1991). Cell-adhesion and growth on polymer surfaces with hydroxyl-groups prepared by water-vapor plasma treatment. Biomaterials.

